# Ceftazidime/Avibactam and Ceftolozane/Tazobactam for Multidrug-Resistant Gram Negatives in Patients with Hematological Malignancies: Current Experiences

**DOI:** 10.3390/antibiotics9020058

**Published:** 2020-02-03

**Authors:** Marianna Criscuolo, Enrico Maria Trecarichi

**Affiliations:** 1 Dipartimento Scienze Radiologiche Radioterapiche ed Ematologiche, Fondazione Policlinico Universitario A. Gemelli, IRCCS, 00168 Roma, Italy; marianna.criscuolo@policlinicogemelli.it; 2 Department of Medical and Surgical Sciences, Unit of Infectious and Tropical Diseases, “*Magna Graecia*” University, 88100 Catanzaro, Italy

**Keywords:** Multidrug-resistant Gram-negative bacteria, Carbapenem-resistant *Klebsiella pneumoniae*, Multidrug-resistant *Pseudomonas aeruginosa*, ceftolozane/tazobactam, ceftazidime/avibactam, bloodstream infections, hematological malignancies

## Abstract

Patients suffering from hematological malignancies are at high risk for severe infections, including in particular bloodstream infections, which represent one of the most frequent life-threatening complications for these patients, with reported mortality rates reaching 40%. Furthermore, a worrisome increase in antimicrobial resistance of Gram-negative bacteria (e.g., cephalosporin- and/or carbapenem-resistant *Enterobacteriaceae* and multidrug-resistant (MDR) *Pseudomonas aeruginosa*) involved in severe infectious complications among patients with hematological malignancies has been reported during the last years. The two novel combination of cephalosporins and β-lactamase inhibitors, ceftolozane/tazobactam and ceftazidime/avibactam, were recently approved for treatment of complicated intra-abdominal and urinary tract infections and nosocomial pneumonia and display activity against several MDR Gram-negative strains. Although not specifically approved for neutropenic and/or cancer patients, these drugs are used in this setting due to increasing rates of infections caused by MDR Gram-negative bacteria. The aim of this review is to describe the actual evidence from scientific literature about the “real-life” use of these two novel drugs in patients with hematological malignancies and infections caused by MDR Gram-negative bacteria.

## 1. Introduction

Among hematologic patients, those affected by acute leukemias and lymphoproliferative diseases are particularly vulnerable. Considering the impairment of the immune system due to underlying malignancies and frequent neutropenia following chemotherapy, they are at high risk for severe infections, including in particular bloodstream infections (BSIs), which represent one of the most frequent life-threatening complications for these patients, with reported mortality rates reaching 40% [1,2,3,4]. While Gram-positive bacteria represented the most frequent etiological agents of bacterial BSIs in patients with hematological malignancies until the last century, during the last decades a clear epidemiological shift has been reported, with Gram-negatives becoming the most prevalent cause [5,6]. Moreover, a worrisome increase in antimicrobial resistance of Gram-negative bacteria (e.g., cephalosporin- and/or carbapenem-resistant *Enterobacteriaceae* and multidrug-resistant (MDR) *Pseudomonas aeruginosa*) involved in severe infectious complications among patients with hematological malignancies has been recently reported [3,4,5,6]. Due to scheduled recurrent and often prolonged hospital admissions, colonization and possible subsequent infections by MDR bacteria have been increasingly reported in the specific setting of hematological patients, particularly in countries with high MDR bacteria prevalence [1,3,6,7,8,9].

Receipt of chemotherapy/radiation therapy has been found as an independent risk factor for carbapenem-resistant *K. pneumoniae* BSI development among patients displaying previous rectal carriage [10]. Moreover, in a prospective observational multicenter study among hematological patients screened at admission, a rate of 6.5% MDR colonization was found. Among these patients, 15.9% (23/144) developed a BSI caused by the same previously identified colonizing pathogen [9]. Another study found male sex and mucositis as predictor of carbapenem-resistant *Enterobacteriaceae* infections subsequent to rectal colonization [11]. In a multicenter study conducted among hematopoietic stem cell transplant recipients, colonization by MDR Gram-negative bacteria predicted a subsequent infection in 30% cases [8].

In general, several studies have reported significantly higher mortality rates among patients suffering from hematological malignancies and BSIs due to MDR Gram-negative bacteria compared to those with BSIs due to susceptible strains, and often, antimicrobial resistance by bacterial isolates and/or the related inadequacy of empirical antimicrobial treatments have been demonstrated among the most important independent risk factors for mortality [1,2,3,4,5,6,12,13,14].

Two novel combination of cephalosporins and β-lactamase inhibitors were recently approved by Food and Drug Administration and European Medicines Agency for treatment of complicated intra-abdominal and urinary tract infections and nosocomial pneumonia. In Table 1, the main characteristics of these two combined antibiotics are reported. Ceftolozane/tazobactam is a combination of the fifth-generation cephalosporin ceftolozane and the β-lactamase inhibitor tazobactam. This combination displays a potent activity against *P. aeruginosa*, including MDR isolates, because it is not removed by efflux pumps and displays a higher affinity with penicillin-binding proteins of cell membrane, in addition to stability against extended-spectrum β-lactamases (ESBLs) and overexpression of AmpC. However, ceftolozane/tazobactam has no activity against carbapenemases [15,16,17,18,19,20,21,22,23].

Ceftazidime/avibactam is a combination of the novel non-β-lactam β-lactamase inhibitor avibactam and ceftazidime. Avibactam prevents the hydrolysis of ceftazidime by many enzymes, including Ambler class A, C and D β-lactameses (e.g., ESBLs, AmpC, *K. pneumoniae* carbapenemases (KPCs) and OXA-48), thus restoring its activity against bacterial strains that produce these enzymes [24,25].

Although not specifically approved for neutropenic and/or cancer patients, these drugs are currently used in these settings due to increasing rates of infections caused by MDR Gram-negative bacteria. The aim of the current review is to describe the actual evidence from scientific literature concerning the “real-life” use of these two novel drugs in patients suffering from hematological malignancies with infections caused by MDR Gram-negative bacteria.

## 2. Ceftolozane/Tazobactam

Severe infections, in particular BSIs, caused by MDR *P. aeruginosa* have been increasingly described among hematological patients, with reported mortality rates of up to 40% [1,26,27,28]. Furthermore, patients suffering from hematological malignancies have been reported to be at highest risk for such infections among neutropenic patients and for inadequate empirical antibiotic therapy, which has been frequently found as one of the most important independent risk factors for mortality [27,29,30,31].

Polymyxins, especially colistin, have been increasingly used for the treatment of infections caused by MDR *P. aeruginosa* during the past years because they often represent one of the few (or the only) treatment options in the setting of patients with hematological cancer. However, many concerns have been raised concerning the use of these drugs, due to high risk of nephrotoxicity and/or neurotoxicity, lack of solid pharmacokinetic/pharmacodynamic data with risk of suboptimal concentrations and recent reported increasing rates of resistance [32,33]. Some studies have been focused on comparing colistin vs. other classes of antibiotics (mainly β-lactams or fluoroquinolones) in treatment of infections caused by MDR *P. aeruginosa* among hematological or solid cancer patients and no difference in clinical efficacy as well as safety was reported [34,35].

After FDA approval and marketing, due to its potent activity against *P. aeruginosa* (including MDR) strains collected worldwide, ceftolozane/tazobactam has been reported in “real-life” general population experiences as clinically effective as well as safe in the treatment of multiple types of MDR *P. aeruginosa* infections [36,37,38,39]. Recently, Pogue et al. conducted a retrospective, multicenter, observational cohort study comparing patients treated with ceftolozane/tazobactam to those who received polymyxin or aminoglycoside-based mono- or combination regimens for treatment of MDR *P. aeruginosa* infections. In their study, authors demonstrated that receipt of ceftolozane/tazobactam was independently associated with a better clinical cure and a lower rate of acute renal injury (adjusted odds ratio 2.63 (1.31–5.30) and 0.08 (0.03–0.22], respectively) [40]. In the specific setting of onco-hematological patients, a recent study conducted in Poland showed that, although they were few, 100% (9/9) of clinical carbapenem-resistant *P. aeruginosa* isolates displayed susceptibility to ceftolozane/tazobactam [41].

From a clinical point of view, “real-life” experiences in the use of ceftolozane/tazobactam in treating MDR *P. aeruginosa* infections in onco-hematological patients have been recently reported. The main clinical and microbiological data described are shown in Table 2. Hakki et al. reported a series of six patients suffering from acute leukemias treated with ceftolozane/tazobactam as monotherapy for MDR *P. aeruginosa* infections, including BSI (three cases), pneumonia (three cases) and soft tissue infection (one case). Although all patients achieved an overall favorable clinical response and lack of toxicity, two patients had a recurrence of non-bacteriemic infections. In one of these two case, the isolated *P. aeruginosa* strain displayed in vitro resistance to ceftolozane/tazobactam; of note, all patients received a dosage of 9 grams (g)/24 h administered as 3 g every 8-h intermittent infusions [42]. Fernández-Cruz et al. conducted a case-control study involving 19 hematological patients treated with ceftolozane/tazobactam and 38 with other antibiotic regiments (including piperacillin/tazobactam, cefepime, ceftazidime, meropenem, ciprofloxacin, colistin or amikacin). There were no differences regarding underlying malignancy, source of infections or neutropenia, but cases compared to controls had infections more likely due to an extensively-drug resistant (XDR) *P. aeruginosa* strain and less frequently involving the blood stream. At 14 days, rates of clinical recovery were comparable between cases and controls (89.5% vs. 71.1%), but 30-day mortality was significantly lower among cases compared to controls (5.3% versus 28.9%) in both the univariate and multivariate analyses. Three patients experienced recurrent central venous catheter-related infections, in at least two cases were due to inadequate source control, but no resistance to ceftolozane/tazobactam was detected in recurrent isolates [43]. Aitken et al. described the case of a 9-year-old boy suffering from relapsed/refractory acute myeloid leukemia and episodes of prolonged neutropenia subsequent to chemotherapy during which the patient experienced recurrent episodes of BSI caused by the *P. aeruginosa* isolates which progressively displayed multidrug-resistance after multiple courses of therapy with different antibiotics. Interestingly, in the last episode, the *P. aeruginosa* isolate displayed resistance (and clinical failure) to ceftazidime/avibactam and a Minimal Inhibitory Concentration to ceftolozane/tazobactam of 8 μg/mL. The patient was therefore successfully treated with a slightly lower dose of ceftolozane/tazobactam (40 mg/kg of ceftolozane component) with a frequency of 3-hour infusion four times a day in combination with tobramycin and ciprofloxacin, to which the isolate displayed in vitro susceptibility [44]. Finally, So at al. reported the case of a patient diagnosed with acute myeloid leukemia who experienced an episode of breakthrough BSI due to a *P. aeruginosa* strain which became resistant to ceftolozane/tazobactam after a prolonged course of therapy. After evidence of early clinical and microbiological cure of BSI obtained by empirical use of combination of ceftolozane/tazobactam and tobramycin, the authors demonstrated in vitro synergistic effects of this combination, which reduced the MIC of ceftolozane/tazobactam from ≥256 to 16 mg/L and the MIC of tobramycin from 4 to 1 mg/L. Therefore, the patient was successfully treated with this antimicrobial combination [45].

## 3. Ceftazidime/Avibactam

The recent introduction of ceftazidime/avibactam has improved the outcomes of infections caused by *Enterobacteriaceae*, which display resistance to carbapenems because of production of class A β-lactamases, including KPCs, class C β-lactamases, and certain oxacillinases (i.e., OXA-48 carbapenemases). Although there are no data derived from randomized trials specifically focusing on clinical efficacy of ceftazidime/avibactam in treatment of infections caused by carbapenem-resistant Gram-negative bacteria, different “real life” experiences demonstrated that treatment with ceftazidime/avibactam has been associated with higher rates of clinical success and survival compared to other antibiotic regimens in treatment of severe infections caused by carbapenem-resistant *Enterobacteriaceae* [46,47]. In particular, Tumbarello et al. conducted a retrospective cohort study including a total of 138 patients with KPC–producing *K. pneumoniae* infections treated with ceftazidime/avibactam as salvage therapy. The overall mortality rate at 30 days was 34.1%. When 104 bacteremic patients treated with ceftazidime/avibactam were compared to an equal number of matched controls who had received salvage therapy regimens that did not include ceftazidime/avibactam, the authors found a significant difference in the 30-day mortality between the two groups (36.5% vs 55.8%) in favor of treatment with ceftazidime/avibactam-based regimes. Moreover, in multivariate analysis treatment with ceftazidime/avibactam was the only predictor of survival. Interestingly, 19/138 (13.7%) patients included in this cohort suffered from hematological malignancies and 15/138 (10.9%) were neutropenic [48].

Clinical experience on use of ceftazidime/avibactam in the specific setting of patients with hematological malignancies is to this date overall scarce; the main clinical and microbiological information contained in the studies currently published to this regard are summarized in Table 2.

Caston et al. have conducted a retrospective multicenter study including 31 patients with hematological malignancies and BSIs caused by carbapenemase (KPC or OXA-48) producing *Enterobacteriaceae*; eight of these patients were treated with directed combination drug regimens including ceftazidime/avibactam and 23 with other antibiotic therapies. The clinical cure rate at 14 days was significantly higher in the former group compared to the latter (75% versus 34.8%), despite mortality at 30 days was not significantly different (25% versus 52.2%), possibly due to a small sample population size [49].

Furthermore, a series of three patients suffering from leukemia with BSIs caused by carbepenem-resistant Gram-negative bacteria (two *K. pneumoniae* and one *P. aeruginosa*) treated with ceftazidime/avibactam as salvage therapy (compassionate use) was reported. Two out of three patients with persistent grade III neutropenia and clinical symptoms of sepsis recovered early after the beginning of definitive therapy, whereas one patient, with septic shock at clinical presentation, after 12 days of treatment and resolution of fever, died of respiratory failure and massive bleeding [50].

Ceftazidime/avibactam is not active against *Enterobacteriaceae* arbouring metallo-β-lactamases (MBLs; e.g., imipenemase metallo-β-lactamase (IMP) and Verona integron encoded metallo-β-lactamase (VIM) groups). However, in vitro studies suggested a synergistic effect of aztreonam combined with avibactam on MBLs-producing *Enterobacteriaceae* and some clinical experiences on successful use of aztreonam combined with ceftazidime/avibactam in treating infections caused by these bacteria have been reported in general population [51,52]. Hobson et al. have recently reported a case of BSI caused by an NDM-1 and cephalosporinases producing *Morganella morganii* isolate. The patient was a 3-year-old girl suffering from a relapsed lymphoblastic acute leukemia with severe aplasia and grade 4 mucositis. The bacterial strain displayed a MIC 0.016 mg/L for combination of aztreonam and ceftazidime/avibactam. The patient was successfully treated with a 10-day course of this antibiotics’ combination without relapse within the subsequent 6 months period. To the best of our knowledge, no other clinical experiences in use of aztreonam and ceftazidime/avibactam combination have been reported in hematological patients [53].

Recently, resistance to ceftazidime/avibactam has been reported and different mechanisms for the emergence of resistance have been investigated [54]. The first reported mechanism of resistance was attributed to sporadic production of Ambler class B β-lactamases. More recently, different amino acid substitutions in KPC genes (e.g., D179Y in bla_KPC-3_) leading to a higher rate of ceftazidime hydrolysis as well as a combined mechanism of acquired mutation leading to nonfunctional porins and an increased production of KPC-3 through the transposition of different plasmids have been reported as conferring resistance to ceftazidime/avibactam [20,55].

## 4. Conclusions

The increasing burden of severe infections due to MDR Gram-negative bacteria, in particular cephalosporin-and/or carbapenem-resistant *Enterobacteriaceae* and MDR *P. aeruginosa*, greatly impacts the prognosis of patients with hematological malignancies, due to the severity of these infections and to delay in appropriate antimicrobial therapy [3,27,56,57]. The two novel antibiotics, ceftolozane/tazobactam and ceftazidime/avibactam, have been used in “real-life” experiences for treatment of such infections with reported clinical and microbiological success in patients with hematological malignancies, although data are currently scarce and based mainly on case series and case reports. More studies are necessary to better define the role of these drugs in hematological cancer patients, for example in empirical use. Novel antibiotics displaying activity against carbapenem-resistant Gram-negative bacteria (e.g., imipenem/relabactam, plazomicin and cefiderocol) are currently under clinical evaluation. Moreover, the new carbapenem/β-lactamase inhibitor meropenem/vaborbactam (with in vitro activity similar to ceftazidime/avibactam but with some peculiarities) was approved by the FDA in 2017 for complicated urinary tract infections (cUTIs) and more recently by the EMA for cUTIs, complicated intra-abdominal infections, hospital-acquired, including ventilator-associated, pneumonia and infections due to aerobic Gram-negative organisms in adult patients with limited treatment options. Of note, eravacycline and cediferocol display efficacy against carbapenem-resistant *Acinetobacter baumannii* isolates. Considering the reported prevalence of infections due to MDR Gram-negatives and the emergence of resistance to ceftolozane/tazobactam and ceftazidime/avibactam, these potential new treatment options, although not specifically investigated in this setting, should be considered in the future also in patients suffering from hematologic malignancies [33,58]. As therapeutic options for infections caused by MDR Gram-negative bacteria are limited, the development of infection prevention strategies is of paramount importance, particularly in high-risk patients such as those with hematological malignancies.

## Figures and Tables

**Table 1:** Main characteristics of ceftazidime/avibactam and ceftolozane/tazobactam.

Antibiotic Combination	Mechanism of Action	Main Targets	Drug Resistance	Dosage (CrCL >50 mL/min)	Approved Clinical Indications	References
**Ceftolozane/Tazobactm**	*Ceftolozane*: inhibition of cell-wall synthesis via binding of PBPs with greater affinity for PBPs 1b, 1c, 2 and 3. *Tazobactam*: inhibition of most Ambler class A and some class C β-lactamases.	ESBL producing Enterobacteriaceae; MDR *Pseudomonas aeruginosa*	Carbepenem-resistant *Enterobacteriaceae*; Carbapenem-resistant *Acinetobacter baumannii*	1.5 g (1 g ceftolozane and 500 mg tazobactam) IV q8h for cIAI I and cUTI 3 g (ceftolozane 2 g and tazobactam 1 g) IV every 8 hours for pneumonia	cIAIs (in combination with metronidazole), cUTIs, Hospital-acquired/ventilator-associated pneumonia	Hong 2013 [15] Giacobbe 2018 [16] Giacobbe 2018 [17] Koulenti 2018 [18] Ho 2019 [19]
**Ceftazidime/Avibactam**	*Ceftazidime*: inhibition of cell-wall synthesis via binding of PBPs with greater affinity for PBP 3. *Avibactam*: inhibition of Ambler class A (e.g., ESBL and KPC), class C (e.g., AmpC), and some class D (e.g., OXA-48) enzymes	Carbapenem-resistant (KPC- or OXA-48 producing) *Enterobacteriaceae*	Ambler class B β-lactamases (i.e metallo β-lactamases) producing Gram-negative bacteria; Carbepenem-resistant *Acinetobacter baumannii*	2.5 g (2 g ceftazidime and 500 mg avibactam) IV q8h	Complicated intra-abdominal infections (in combination with metronidazole), Complicated urinary tract infections, Hospital-acquired/ventilator-associated pneumonia Aerobic Gram-negative infections in patients with limited treatment options (only EMA)	Hong [15] Lagacé-Wiens 2014 [20] Sheu C-C 2019 [21]

PBPs, penicillin-binding proteins; ESBL, extended-spectrum beta-lactamase; MDR, multidrug resistant; IV, intravenous; EMA, European Medicines Agency; KPC, *Klebsiella pneumoniae* carbapenemase; cIAIs complicated intra-abdominal infections; cUTIs complicated urinary tract infections.

**Table 2:** “Real-life” experiences reported in published studies on use of ceftolozane/tazobactam and ceftazidime/avibactam for treatment of severe infections caused by MDR Gram-negative bacteria in patients with hematological malignancies.

Antibiotic Combination	Type of Study	N of Patients Treated with C/T or C/A	Year of Interest	Isolated Species	Clinical Sample Sites	Combination Therapy	Mortality	Recurrence	Resistance
Ceftolozane/Tazobactam									
Hakki 2018 [42]	Retrospective case series	6	NA	*MDR P. aeruginosa*	Blood, BAL, soft tissue	None	30-day 0%	1 case	1 case
Fernández-Cruz 2019 [43]	Retrospective case-control	19	2016–2018	*P. aeruginosa* (MDR 51.2%)	Blood, BAL, soft tissue, urine	42.1% (amikacin, levofloxacin, colistin, fosfomycin)	30-day 5.3%	3 cases	None
Aitken 2016 [44]	Case report	1	NA	*MDR P. aeruginosa*	Blood	Tobramycin and ciprofloxacin	0	No	None
So 2019 [45]	Case report	1	NA	Ceftolozane/tazobactam *P. aeruginosa*	Blood	Tobramycin	0	No	Yes
Ceftazidime/Avibactam									
Caston 2017 [49]	Retrospective	8	2012–2016	Carbapenemase-producing *Enterobacteriaceae*	Blood	100% (aminoglycoside, carbapenems, fosfomycin, tigecycline and/or colistin)	30-day 25%	None	None
Metafuni 2019 [50]	Case series	3	2017–2018	Carbapenemase-producing *K. Pneumoniae* (2) MD*R P. aeruginosa* (1)	Blood	100% (carbapenems, tigecycline, colistin)	30-day 33.3%	None	None
Hobson 2019 [53]	Case report	1	NA	NDM-1-Producing *Morganella morganii*	Blood	Aztreonam	0	None	None

C/T, ceftolozane/tazobactam; C/A, cefzidime/avibactam; NA, not available; MDR, multidrug-resistant; BAL bronchoalveolar lavage.
